# The Amplitude and Inactivation Properties of the Delayed Potassium Currents Are Regulated by Protein Kinase Activity in Hair Cells of the Frog Semicircular Canals

**DOI:** 10.1371/journal.pone.0067784

**Published:** 2013-07-02

**Authors:** Marta Martini, Rita Canella, Riccardo Fesce, Maria Lisa Rossi

**Affiliations:** 1 Dipartimento di Scienze della Vita e Biotecnologie, Ferrara University, Ferrara, Italy; 2 Centre of Neuroscience and DISTA, Insubria University, Varese, Italy; University of South Florida, United States of America

## Abstract

In hair cells dissected from the frog crista ampullaris, the combination of a calcium-dependent (IKCa) and a purely voltage-dependent component (IKV) gives rise to the delayed potassium current complex (IKD). These currents have been recently reported to display slow depolarization-induced inactivation and biphasic inactivation removal by hyperpolarization. The amplitude and inactivation kinetics of both IKCa and IKV are drastically modulated by a previously unrecognized mechanism of protein phosphorylation (sensitive to kinase inhibitors H89 and KT5823), which does not interfere with the transient potassium current (IA) or the calcium current (ICa). IKD amplitude was stable in cells patched with pipettes containing 8 mM ATP or under perforated-patch; under these conditions, a 10 min treatment with 10 µM H89 or 1–10 µM KT5823 reduced IKD amplitude by a mean of 67% at +40 mV. Similarly affected was the isolated IKV component (ICa blocked with Cd^2+^). Thus, a large potassium conductance can be activated by depolarization, but it is made available to the cell to a variable extent that depends on membrane potential and protein kinase activity. The total gKD ranged 4.6–44.0 nS in control cells, according to the level of steady-state inactivation, and was reduced to 1.4–2.7 nS after protein kinase inhibition. When sinusoidal membrane potential changes in the −70/−10 mV range were applied, to mimic receptor response to hair bundle deflection, IKD proved the main current dynamically activated and the only one regulated by PK: H89 decreased the total outward charge during each cycle by 60%. Phosphorylation appears to control both the amount of IKCa and IKV conductance activated by depolarization and the fraction thereof which can be rescued by removal of inactivation. The balance between the depolarizing transduction current and the repolarizing potassium current, and eventually the transmitter release at the cytoneural junction, are therefore modulated by a phosphorylation-mediated process.

## Introduction

Inactivation is a typical property of ion channels and determines, in a voltage and time-dependent manner, the number of channels which are available for activation at any moment. The kinetics, voltage- and time-dependence of inactivation onset and removal are quite different, for the various conductances, so that the cell response is molded by the previous history of membrane potential. In the hair cells of the frog semicircular canal virtually all conductances are influenced by membrane potential, independent of biochemical and receptor transduction processes. For example, the IA activates in response to voltage shifts within a few milliseconds, and similarly fast is the onset of current inactivation [1]. On the other hand, inactivation is removed by a mirroring – though relatively slower – voltage-dependent process. An unexpected and more complex set of mechanisms of inactivation (onset and removal) have been recently described for the delayed potassium current complex, IKD, in frog hair cells [1]. The membrane potential level modifies by several-fold the amplitude of the current evoked by depolarization; a sojourn of a few tens of milliseconds at a given holding level is sufficient to markedly modify the amplitude of the response. At difference with IA, the inactivation process is quite slow and only partial for IKD. Thus, the intensity of the current upon activation is influenced by previous conditioning, but the changes are much more persistent over time.

Two relevant experimental manipulations strongly interfere with potassium currents: the ionic-metabolic modifications of the internal cellular milieu which occur in patch-clamp experiments and the concentration of ATP in the pipette. In hair cells, ICa is particularly sensitive to such manipulations, in that its amplitude exhibits relevant up- or down-regulation, according to the internal ATP levels artificially imposed [2], [3].

Less clear, at least in semicircular canal hair cells, is whether currents might also be modulated by intracellular mechanisms related to second messenger systems. Activation of protein kinases, linked to various signaling pathways, has been shown to dynamically modulate ionic channel properties in excitable cells; this may entail major modifications of the excitability machinery (see, for example, the reviews by Jonas and Kaczmareck [4] and Park et al. [5]). Among the targets of protein kinase modulation, potassium channels have deserved special attention. The phosphorylation state of the channel-protein has been shown to affect the open-probability of the channel (Po) (for Kv2.1: Park et al. [6]; for Kir6.2: Light et al. [7]), the inactivation mechanism (for Kv4.2: Jerng et al. [8]; for Kv3.4: Covarrubias et al. [9]; for Kv1.4: Roeper et al. [10]), the current activation curve and the calcium affinity (for calcium-dependent channels of BK type [11]), the total number and clustering of channels in the plasma membrane as a consequence of relocation of the channel protein [12], [13].

Given the sensitivity of ionic currents to intracellular ATP levels, in isolated frog hair cells, in this study we explore the effects of treatments potentially involved in phosphorylation of the most relevant channels. After considering the effects on individual isolated currents, the dynamic interplay of the single currents is analyzed, in generating the integrated electrical behavior of the cell. Finally, a minimal electrical model of the IA current in the isolated hair cell is proposed, which might help to understand the complex current interplay during sensory stimulation.

The mechanisms that modulate ion channel properties have diversified voltage dependence and operate on quite different time scales, thereby producing a wide activity-dependent regulation of hair cell excitability.

## Materials and Methods

### Preparation of Hair Cells

Frog labyrinth preparation, stimulation and recording procedures have been described in detail previously [1]–[3], [14]. All procedures for handling of animals and surgery were approved by the Animal Care and Use Committee of the University of Ferrara. The experiments were performed on wild frogs (*Rana esculenta*, 25–40 g body weight) that were mainly harvested in summer. The animals were anesthetized by immersion in tricaine methane sulphonate solution (1 g/l in water) and subsequently decapitated. Dissection of the two labyrinths was performed in a solution of the following composition (mM): 120 NaCl, 2.5 KCl, 0.5 EGTA, 5 4–2-hydroxyethyl-1-piperazineethanesulfonic acid (HEPES), 3 glucose and 20 sucrose. The final pH was 7.2 and the osmolality 260 mOsmol/kg. Six ampullae were treated for a period of 20–30 s with subtilisin A, type VIII (50 µg/ml, Sigma); the protease was thereafter blocked by trypsin inhibitor type II-S (Sigma) added to the dissection solution (final concentration, 0.7 mg/ml). Ampullae were then transferred to the experimental chamber (500-µl volume), submerged in the standard extracellular solution (mM: 120 NaCl, 2.5 KCl, 2 CaCl_2_, 1 MgCl_2_, 5 glucose, Tris buffer, pH 7.3–5) in the presence of the trypsin inhibitor. Hair cells were mechanically dissociated from the ampullae by gently scraping the epithelium with a fine forceps. The protease inhibitor was eventually washed out before starting with electrophysiology. The glass bottom of the experimental chamber was coated with chloro-tri-n-butyl-silane to prevent cell sticking.

Cells were viewed through a TV monitor connected to a contrast enhanced video camera (T.I.L.L. Photonics, Planegg, Germany) and continuously superfused with the extracellular solution. The camera was coupled to an inverted microscope (Olympus IMT-2, Tokyo, Japan) equipped with a 40× Hoffman modulation contrast system.

### Cell Recording and Solutions

Macroscopic currents were recorded at room temperature, within 2 h after cell dissociation, by using the patch-clamp technique (EPC-7, List-Electronic, Darmstadt, Germany), usually in the ‘whole-cell’ configuration. Pipettes were pulled from 50-µl glass capillaries (Drummond, Broomall, PA) and fire-polished to a pipette resistance of 3–4 MΩ. The pipette was filled with the following (mM): 110 KCl, 2 MgCl_2_, 0–8 ATP (K salt), 0.1 GTP (Na salt), 5 EGTA-NaOH, 10 HEPES-NaOH (pH 7.2; 235 mOsmol/kg). [K^+^]_i_ was maintained constant when K-ATP levels varied. When calcium currents were measured, the pipette solution composition was (mM): 90 CsCl, 20 TEA-Cl, 2 MgCl_2_, 8 K-ATP, 0.1 Na-GTP, 10 HEPES, 5 EGTA (pH 7.2; 235 mOsm/kg).

Cd^2+^ (200 µM) was used as a blocker of voltage-dependent calcium channels. It was applied by rapidly changing (typically <50 ms) the external solution by horizontally moving a multi-barrelled perfusion pipette positioned in front of the recorded cell (with a computer-controlled stepping motor). The perforated-patch configuration was obtained by adding amphotericin B (300 µg/ml) to the pipette solution. To facilitate the giga-seal formation, the amphotericin-containing solution was used to backfill the electrode, while the tip was filled with an amphotericin-free solution.

Series resistance ranged between 8 and 22 MΩ. The cell capacitance and series resistance were electronically compensated (50–75%) before each voltage-clamp protocol was run. Leak was measured near resting potential with 10 mV×15 ms hyperpolarizing pulses; this leakage current was subsequently subtracted, assuming a linear behavior, in correcting current recordings offline. Currents were low-pass filtered at 5 kHz and acquired online at 10 kHz with pClamp hardware and software (pClamp 9.1 and Digidata 1322A interface; Axon Instruments, Union City, CA, USA). Data were analyzed offline by using pClamp 9.1 and Matlab 10a (The MathWorks, Natick, MA, USA) software. They were not corrected for the liquid junction potential, estimated to be about +4 mV under our standard recording conditions [1].

N-[2-(p-Bromocinnamylamino)ethyl]-5-iso-quinolinesulphonamide (H-89), 2,3,10,11,12hexahydro-10R-methoxy-2,9-dimethyl-1-oxo-9S,12R-epoxy-1H-diindolo[1,2,3-fg:3′,2′,1′-kl]pyrrolo[3,4-i][1], [6]benzodiazocine-10-carboxylic acid, methyl ester (KT5823), IBMX and Rolipram were dissolved in dimethyl sulfoxide at a final concentration <0.1% and added to the perfusion medium shortly before use. 8-Bromo-cAMP and 8- (4- chlorophenylthio)guanosine- 3′, 5′- cyclic monophosphate (8-pCPT-cGMP) were similarly bath-perfused. All drugs were obtained from Sigma.

The IKD conductance of the cell, and the fraction thereof which is available at any moment, were estimated, assuming linearity of the instantaneous I–V curve, according to the equation: *g*KID = IKD/(V–E_K_), with E_K_ = −95 mV. The peak conductance was evaluated for currents evoked at V = +40 mV, where the N-shaped I–V curve of IKD exhibits its maximum, close to the experimental equilibrium potential of the calcium current.

Previous results indicated that the magnitude and kinetic properties of single ionic currents do not display any consistent differences and/or correlations among the different cell types [1]. Since the basic resting properties of the isolated hair cells that survived the dissection procedures were relatively homogeneous, no attention was paid to the specific cell type in analyzing and comparing different experimental conditions. No systematic differences were produced by the levels of ATP here tested (1 vs. 8 mM) or by external (or internal, not shown) H89 application, in the general electrical properties of hair cells (see Table 1). This rules out that any conductance became activated or inhibited by the treatments in the unstimulated cell.

**Table 1:** Passive parameters of hair cells used in this study.

	Cell input capacitance pF	Cell input resistance GΩ	Zero current membrane potential mV
Pipette	In control After 6 min saline	In control After 6 min saline	In control After 6 min saline
1 mM ATP	11.3±0.8 10.6±0.8	1.7±0.3 1.3±0.1	−70.0±4.0¶ −65.2±4.0^†^
8 mM ATP	11.3±0.8 11.6±0.8	1.0±0.2 1.2±0.2	−73.9±3.4−72.1±3.5
8 mM ATP+H89*	11.1±0.6 10.7±0.6	1.2±0.1 1.3±0.2	−69.6±5.3−69.2±5.6

* 10 µM H89 was added to the bath after the initial measure in control saline.

¶ −73.0±7.1 and †−68.8±6.7 mV in perforated-patch experiments (n = 7). Otherwise, n = 12, in each group. Mean ± SE.

### The IA Model

The transient outward conductance, *g*_A_, in the frog hair cell was assumed to obey a Hodgkin-Huxley paradigm, controlled by a pair of voltage-dependent parameters, *a* and *h_a_*, respectively related to activation and inactivation. IA was modeled according to the kinetic scheme: IA(*V*,t) = g_A_·*a*(*V*,t)·*h_a_*(*V*,t)·(*V*_m_–E_K_), where g_A_ is the maximal conductance (20.2 nS per cell), in the absence of inactivation, *V*_m_ is the membrane potential, and E_K_ is the potassium ion equilibrium potential (−95 mV). The steady-state values and the time constants of the activation and inactivation parameters are presented as functions of voltage, and are valid within the voltage range of physiological interest (−75/0 mV). The complete set of equations describing the voltage dependence of the activation and inactivation variables are derived from the experimental data presented in a previous report (Martini et al. [1]) and are as follows:
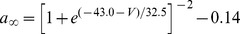


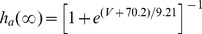


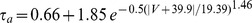


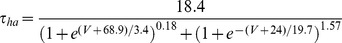



### Statistical Analysis

All data are reported in the text and in the figures as means±SE. Comparisons among different conditions were performed by Student’s *t*-test (paired *t*-test for paired observations) or by ANOVA. Values of *P*<0.05 were considered significant.

## Results

**Figure 1:**
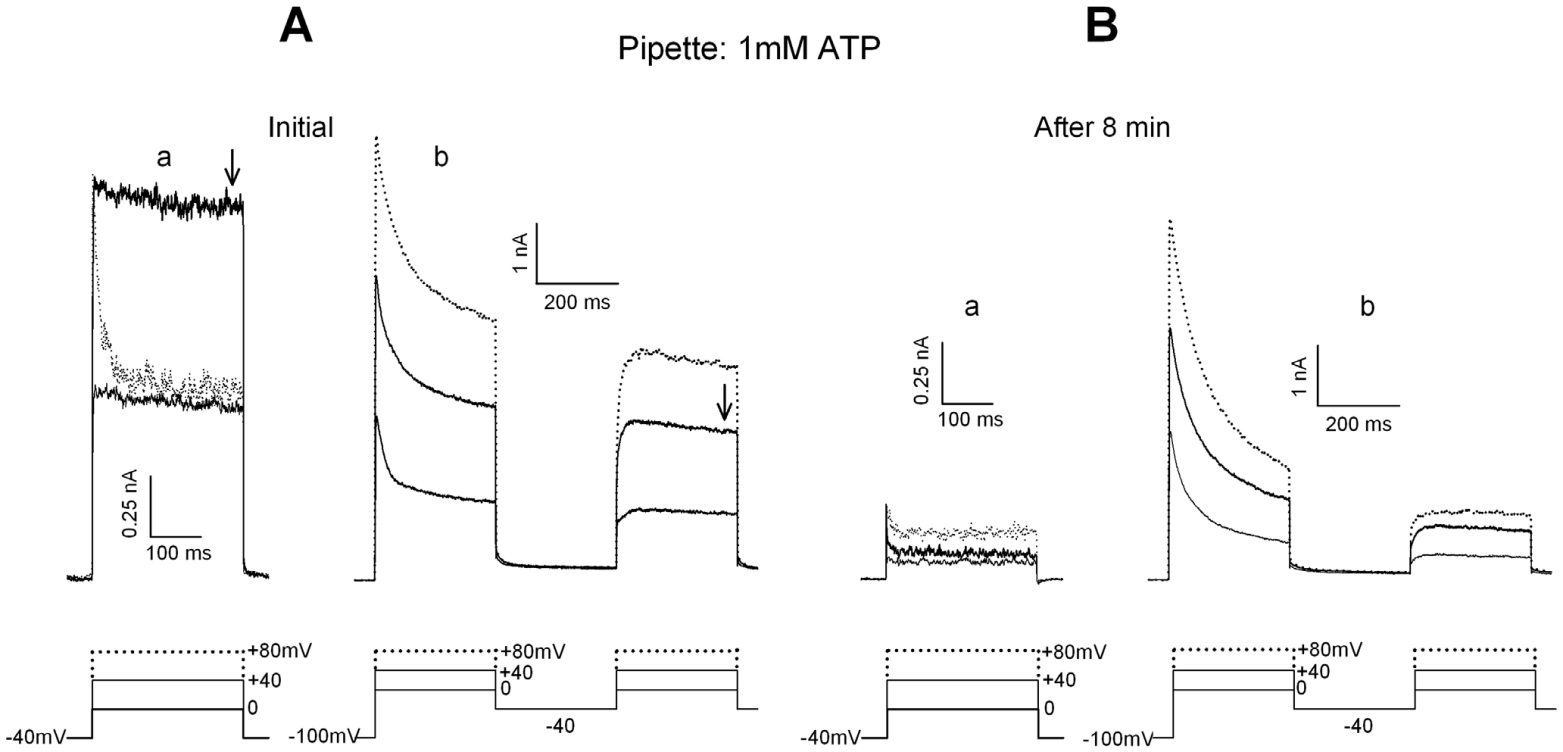
Decomposition of the effects of long-lasting recording with 1 mM ATP-patch clamp-pipettes. **A.** Initial tracings of the currents evoked in a hair cell by steps to 0/+80 mV (only three steps illustrated for clarity) from a holding potential of −40 mV (**a**) and by the same protocol after a 1 sec conditioning at −100 mV (**b**). In (**b**) the current families are evoked twice: immediately following conditioning (IA inactivation removed) and after a sojourn at −40 mV sufficient to maintain full IA inactivation (but with little effect on the removal of IKD inactivation by the previous conditioning at −100 mV). The difference-currents between the two episodes dissect the pure IA at each test potential (see text and Martini et al. [1], for details). Arrows indicate the IKD amplitude used to evaluate the effect of inactivation removal. **B.** Same protocols as in **A**, applied in the same cell 8 min later. Note the drastic decrease in IKD amplitude and the monotonic increase in the I–V curves (as opposed to panel **A**). IA peak amplitude, after appropriate dissection, remains constant at each test potential, the apparent decrease being exclusively due to the IKD component.

Typical ionic currents recorded in hair cells acutely isolated from frog semicircular canals are illustrated in Fig. 1, together with the voltage protocols that explore their main features: voltage-dependent activation, isolation of the single current components and inactivation mechanisms. The pipette K-ATP complement was 1 mM. The compound delayed K current, IKD, is displayed in panel Aa, as evoked from a holding level of −40 mV (to inactivate the transient IA potassium current). IKD is a compound current contributed to by the calcium-dependent potassium current, IKCa, the purely voltage-dependent potassium current, IKV, and a smaller calcium component, ICa. For clarity, only three tracings from the complete I–V curve are illustrated: the current is well developed during the step to 0 mV; it reaches its maximal amplitude at +40 mV (I–V curve peak); the current amplitude displays a region of decrement at more positive voltage steps, conferring the I–V curve a typical N-like shape (see Fig. 2C in Martini et al. [1]). Fig. 1Ab illustrates the protocol to dissect the IA current in isolation, from the difference between the first response (following a 1-s pre-pulse at −100 mV to remove IA inactivation) and the second response (following a 300 ms sojourn at −40 mV to keep IA inactivated). This procedure also reveals the recently reported inactivation of IKD: in fact, IKD amplitude is smaller at −40 mV holding potential (panel Aa) than the steady response following the hyperpolarizing pre-pulse (arrow in panel Ab, note different amplification). Thus, a 1-s sojourn at −100 not only removes IA inactivation, but is also able to remove a fraction of steady inactivation of IKD (details in Martini et al. [1]). The fold-increase in IKD amplitude, following hyperpolarization, will be referred to as inactivation removal coefficient (*irc*). At each value of membrane potential, the *irc* constitutes an indirect measure of the extent of steady-state inactivation of the channels contributing to IKD. Both protocols were repeated every 2 min during a period of 6–10 min. Fig. 1B illustrates the current modifications that occurred during the first 8 min observation period. They have been systematically observed in 6 cells and can be listed as follows: 1) the delayed potassium currents decreased in amplitude (rundown) and ended up displaying a different I–V shape: the N-type profile was no more present in panel Ba (larger response at +80 mV than at +40 mV); 2) the IA current was not affected by any rundown: its peak amplitude remained virtually constant (4.1 vs. 4.4 nA at +80 mV; 1.5 vs 1.9 nA at 0 mV); 3) the inactivation machinery of IKD was operating throughout the observation period (actually, in this example the *irc* increased from the initial value of 1.6 to 7.4 at 8 min); 4) the time-course of IKD response was very similar, independent of the recording time; in particular, activation was very fast when the current was evoked from −40 mV holding potential, but much smoother after inactivation removal, at all times. Since ICa often runs down during long-lasting recordings with 1 mM internal ATP [2], some of the effects might be related to a decrease in the IKCa component of IKD.

**Figure 2:**
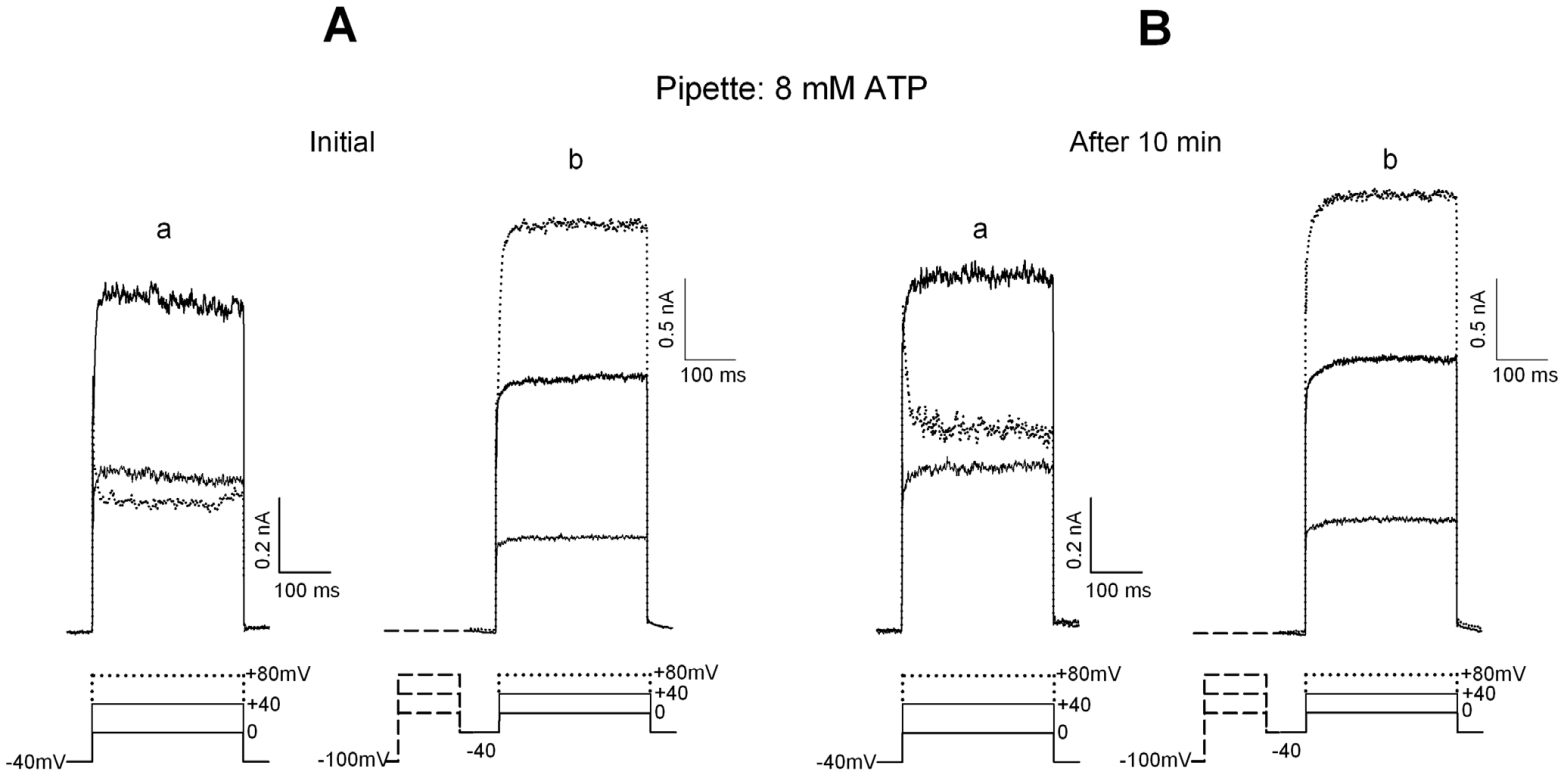
The restoring effect of ATP on delayed potassium currents. **A and B.** The evoked currents are recorded with 8 mM ATP in the pipette, under the same conditions as in Fig. 1. The tracings corresponding to the conditioning period at −100 mV and the subsequent IA+IKD responses are not shown in **Ab** and **Bb**, for clarity. Note that both IKD peak amplitude and the efficiency of inactivation removal are preserved over the whole observation period.

### Stabilizing Effect of ATP

The amplitude of IKD current is liable to run-down, independent of inactivation removal. Since steady-state conditions of the IKD machinery are a prerequisite for a reliable evaluation of the treatments, it seemed important to establish recording conditions such that IKD properties remained stable over time. The stabilizing action of high intracellular ATP concentration on ionic current amplitude was verified first. Alternatively, the ‘perforated-patch' configuration was used to minimize the perturbation of the internal milieu inherent with ‘whole-cell' recording. In Fig. 2 the effects of 8 mM K-ATP in the pipette are shown, in experiments mirroring those of Fig. 1 (the first response, containing IA, has been omitted in both “b” protocols, for clarity). The delayed current complement clearly remains constant during a 10-min observation period: the I–V curve is virtually identical, its N-type shape is preserved throughout, and the inactivation removal mechanism is remarkably constant, with an *irc* value around 1.8. Similar results were observed when the perforated whole-cell configuration was tested (see below). High ATP in whole-cell was generally preferred here to the perforated-patch configuration, in order to clamp the internal ATP at a known concentration; moreover, high ATP was previously shown to prevent ICa amplitude rundown in hair cells and to preserve it from inactivation even during prolonged depolarization [3]. Despite the powerful stabilizing effect of ATP, the rundown of IKD was not reversed when pipettes back-filled with high ATP, but with 1 mM ATP at their very tip, were used. Cell loading with ATP was thus delayed by the time required for high ATP to equilibrate within the pipette: the usual current decay started to take place; in 3 out of 5 cells, the current decay broke off, but it did never reverse.

**Figure 3:**
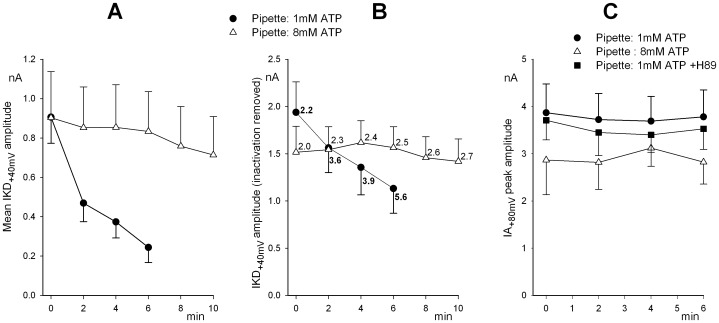
The effect of a variable internal ATP concentration on potassium current properties during long-lasting recording. **A.** Time course of mean IKD amplitude evoked at +40 mV, recorded with pipettes filled with 1 mM ATP (filled circles; n = 6) or 8 mM ATP (triangles; n = 9). Holding potential was −40 mV throughout. **B.** Mean IKD amplitude after inactivation removal at −100 mV for 1 s in the same cells as shown in **A**. Numbers close to symbols indicate the ratio of current amplitude after/before inactivation removal (*irc*). **C.** Mean peak amplitude at +80 mV of pure IA, after current dissection as illustrated in Fig. 1 and text, in cells recorded with 1 mM ATP-pipettes (filled circles; n = 7), 8 mM ATP-pipettes (triangles; n = 5) or 1 mM ATP pipettes with 10 µM H89 added to the bath (squares; n = 6).

Figure 3 summarizes the mean behavior of currents in the presence of 1 or 8 mM ATP in the patch pipette (6 and 9 cells, respectively). Current amplitudes evoked at +40 mV are presented; this single point is representative of the whole I–V curve, since the action of ATP was equally evident at any membrane potential. Panel A illustrates responses from −40 mV holding potential: the IKD current amplitude decays with time in low, but not high ATP. Panel B shows responses after inactivation removal (1 s at −100 mV) in the same group of cells. The numbers associated to each symbol indicate the corresponding values of *irc*. Both IKD amplitude and the *irc* value remained remarkably constant with high internal ATP. Conversely, the amplitude of IKD clearly declined with time in low ATP, even after inactivation removal, in spite of the parallel increase in *irc* (i.e. the rundown was not accounted for by rapidly reversible inactivation alone). Panel C shows that the peak amplitude of IA at +80 mV was totally insensitive to ATP concentration, even over long-lasting recording (n = 5, high ATP; n = 7, low ATP). Insensitivity of IA peak amplitude to H89 treatment was observed not only with 1 mM ATP+H89 in the pipette (Fig. 3C; n = 6), but also when the perforated-patch configuration was used (not shown; n = 6).

The mix of 1 mM ATP plus 7 mM K-ADP in the pipette did not substitute for the 8 mM ATP solution in preventing current rundown.

### Effect of H89, a Protein Kinase Inhibitor, on IKD

**Figure 4:**
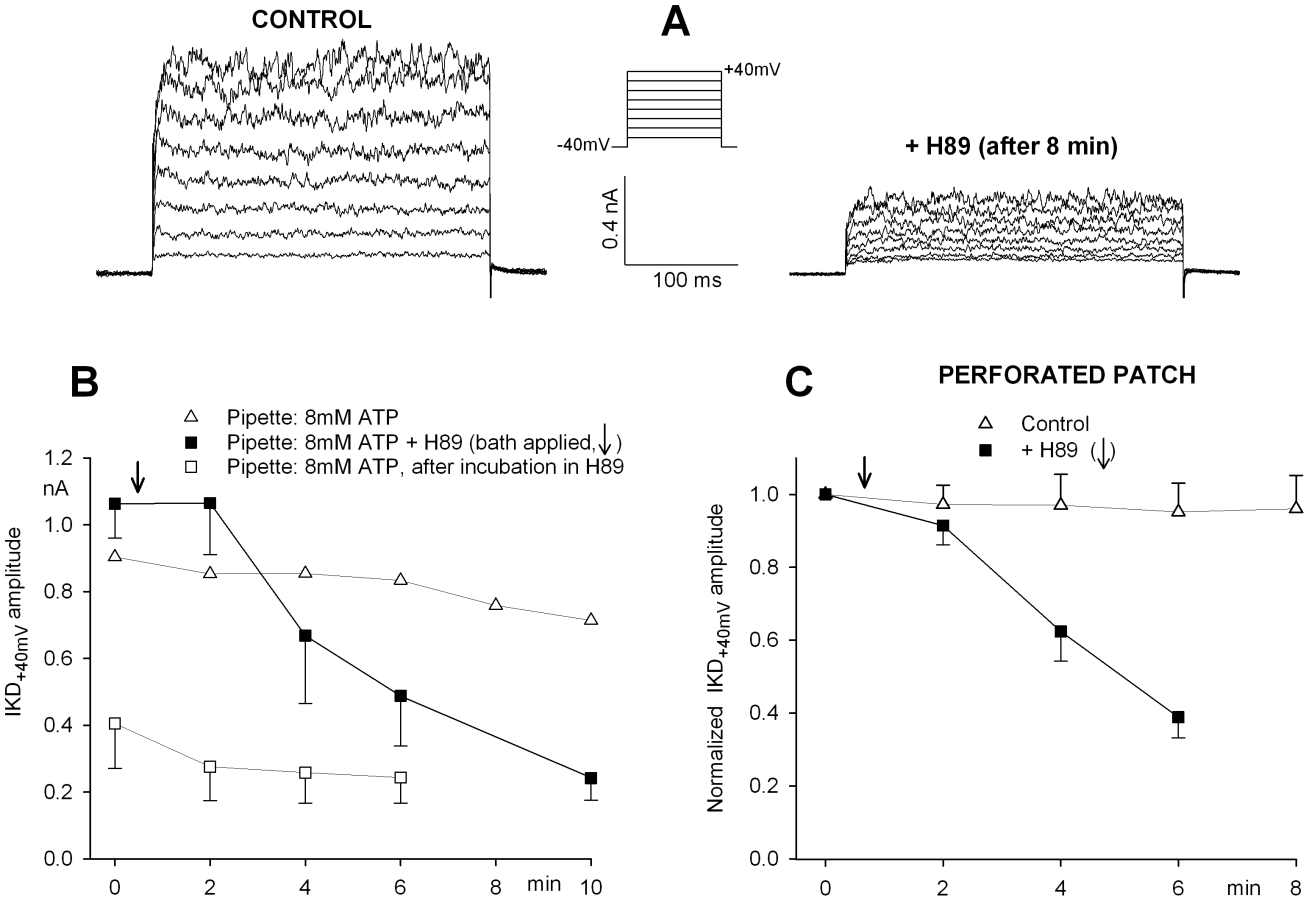
Effect of H89 on IKD amplitude under whole-cell or perforated-patch configuration. **A.** Representative IKD current families evoked in the same cell before and 8 min after H89 bath application. Holding potential was −40 mV, to inactivate IA. **B.** Mean IKD amplitude, evoked at +40 mV from a holding potential of −40 mV, at increasing time in control hair cells (triangles; same data as in Fig. 3**A**), in cells exposed to 10 µM H89, bath-applied at arrow (filled squares; n = 7), and in cells previously exposed to H89 for 15–30 min before starting whole-cell recording (open squares; n = 6). Patch pipette contained 8 mM ATP throughout. **C.** Time course of the normalized mean IKD amplitude, evoked at +40 mV from −40 mV, in control (triangles; n = 7) and cells exposed to 10 µM H89, applied at arrow (squares; n = 6). Perforated-patch recording throughout.

Two protein kinase inhibitors have been tested: 10 µM H89 and 1–10 µM KT5823, presumably specific for PKA or PKG, respectively. H89 was bath applied, under the conditions (8 mM ATP or perforated patch) which have been shown above to stabilize the delayed currents. Figure 4A shows representative IKD tracings, evoked in the same cell over the −30/+40 mV range from a −40 mV holding potential, before and 8 min after H89 treatment. Figure 4B displays the time course of the peak IKD amplitude, evoked at +40 mV from −40 mV holding potential, with 8 mM ATP in the pipette (triangles, same data as in Fig. 3A), and the same measurements in the presence of 10 µM H89, applied to the bath at the arrow (filled squares, n = 7), or pre-incubated for a 15–30 min period (open squares, n = 6). The PKA inhibitor, H89, clearly produced a substantial IKD decay, in cells otherwise stable. Despite the intrinsic variability among single hair cells, the mean initial IKD amplitude was significantly lower in cells pre-incubated in H89 (404.7±134 pA, n = 6) vs. the gross mean of controls (983.1±168pA, n = 16; *P*<0.05, Student’s *t*-test), suggesting that the effects of the treatment were already present when the recording started.

Similar experiments using the perforated-patch configuration yielded overlapping results (Fig. 4C; 7 control vs. 6 H89 treated cells).

**Figure 5:**
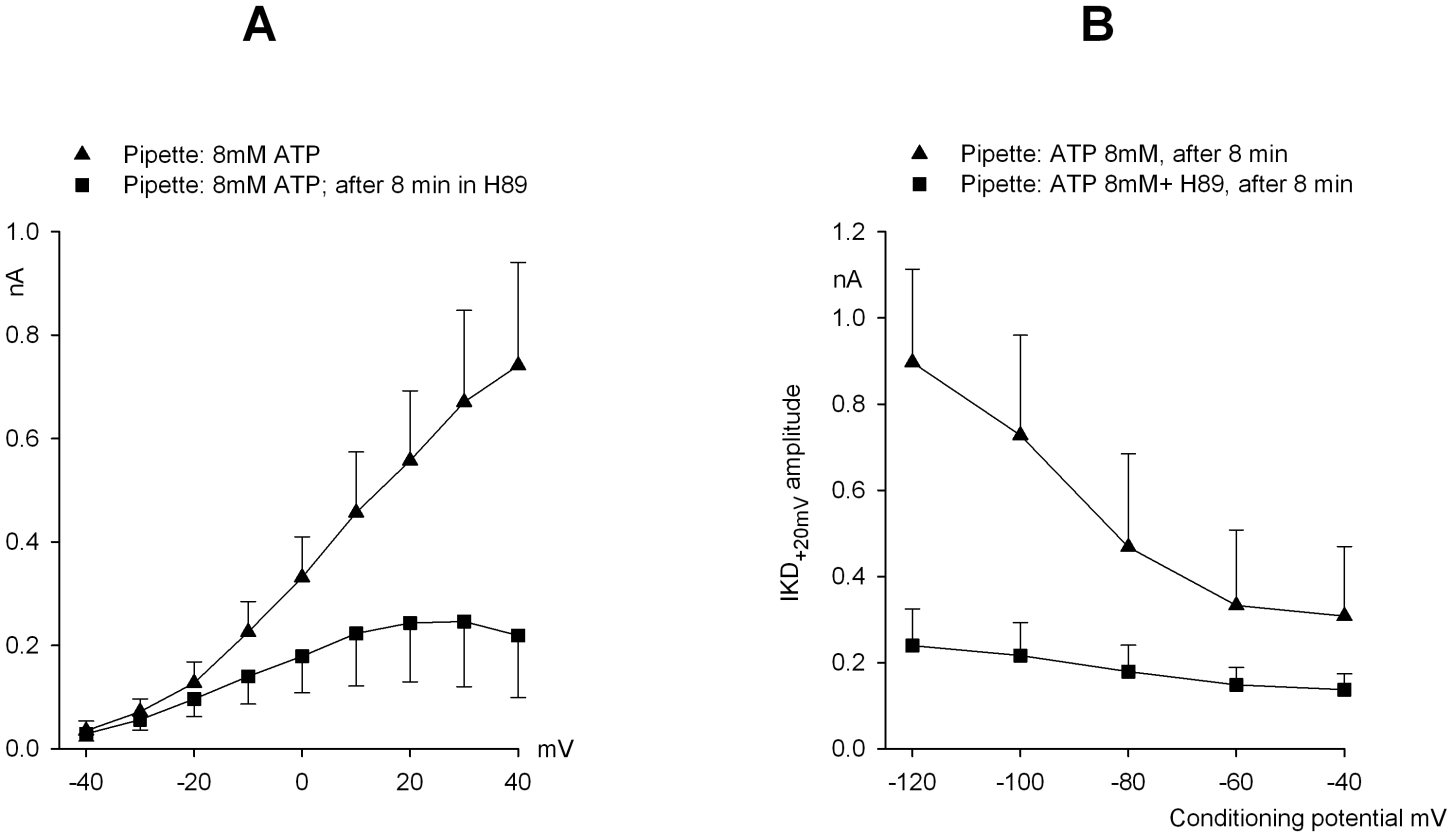
H89 effect on I–V curves of IKD and on IKD inactivation removal. **A.** Mean I–V curves in the same group of hair cells (n = 5) evaluated in normal saline (triangles) and 8 min after application of 10 µM H89 to the bath (squares). **B.** IKD fast inactivation was progressively removed by a 1 s sojourn at increasing conditioning potentials in the range −40/−120 mV, and thereafter tested at +20 mV, in control cells (triangles; n = 6) or hair cells previously exposed for 8 min to 10 µM H89 (squares; n = 8). Each conditioning episode was followed by a 30 s period at −40 mV to allow inactivation to fully develop [1]. See text for the fit of data with Boltzmann-type equations. Pipette with 8 mM ATP throughout.

The mean I–V curves in the same groups of cells, before and after 8 min exposure to H89 (8 mM ATP in the pipette) are shown in Fig. 5A. From these data, some voltage dependence in the effect of H89 is apparent. In particular, the drug appears to more strongly affect currents at potentials positive to +10 mV. The distinct components which comprise the IKD complex (a mix of calcium and potassium currents) intrinsically exhibit different I–V curves, so that the overall effect of H89 on the potassium component alone can be best evaluated close to the equilibrium potential for calcium ion (about +40 mV), where calcium current contribution is minimal.

As mentioned, and clearly evident in Figs. 1 and 2, inactivation of both IA and IKD is readily removed at −100 mV. Actually, the delayed current, which was originally thought to be exempt of inactivation, in addition to fast recovery also displays a well-defined process of slow removal of inactivation. The two processes are readily distinguished: the fast one develops with a time constant of tens of milliseconds, while the slow one only ensues after a delay of about 300 ms and thereafter reaches its maximum with a time constant of seconds [1].

The voltage dependence of the fast, partial removal of IKD inactivation was investigated by conditioning for 1 sec at various potential values (−40/−120 mV voltage range) hair cells either untreated or exposed for 8 min to H89 (8 mM ATP-pipette). In addition to reducing the absolute IKD current, H89 clearly impaired the inactivation process, as shown in Fig. 5B. A 1-s sojourn at different conditioning potentials produced a markedly voltage-dependent removal of inactivation in control cells, whereas in the presence of H89 the inactivation removal I–V relation was almost completely flattened. In particular, when the responses at +20 mV were compared in each cell before and after the sojourn at −120 mV, a mean *irc* value of 5.5±1.7 was obtained in control cells (n = 5), versus a *irc* of 1.7±0.1 following H89 treatment (n = 8; *P*<0.001; Student’s *t*-test). Since an *irc* value >1 was still observed, following H89 treatment, it was of interest to investigate whether not only the magnitude but possibly the voltage dependence as well of the inactivation removal might be affected by H89. Steady-state inactivation is usually expressed by a voltage-dependent “*h*” parameter, which may range from 0 (full inactivation) to 1 (inactivation completely removed). Since inactivation of IKD is only partial, the *h*_KD_ parameter was estimated by measuring peak currents, isolating the fraction subject to inactivation (i.e. the current remaining after −40 mV pre-conditioning was subtracted) and normalizing the results to their largest value (that obtained after −120 mV preconditioning). The voltage dependence of *h*_KD_ was described by Boltzmann-type equations fitting the data, which yielded the following parameters: half-potential, *V1/2* = −88.2±2.1 mV, and slope coefficient, *b = *−10.2±1.5 in control (n = 6) vs. *V1/2 = *−83.8±1.7 mV, *b* = −10.8±1.4 in H89 (n = 10). ANOVA analysis indicated that the slight shift to the right of the *h*_KD_ curve in H89 was not statistically significant. Thus, the extent, but not the voltage dependence, of fast inactivation is affected by pharmacological manipulations of PKA.

The slow component of IKD inactivation also is a likely target of regulatory mechanisms; thus, it was similarly investigated next, in the presence of 8 mM ATP and H89. Slow removal of inactivation was evaluated by applying a 100-s pre-conditioning pulse at −100 mV. This produced an additional consistent increase of the delayed current amplitude evoked by a step to +40 mV, by 62±11% in controls (n = 5) and 16±5% (n = 6) in H89-treated hair cells. The difference was statistically significant (*P*<0.01; Student’s *t*-test), suggesting that both fast and slow components of the process of removal of IKD inactivation are modulated by H89 (i.e., they presumably involve a phosphorylation process).

### Effect of H89 on IKV, IA and ICa

**Figure 6:**
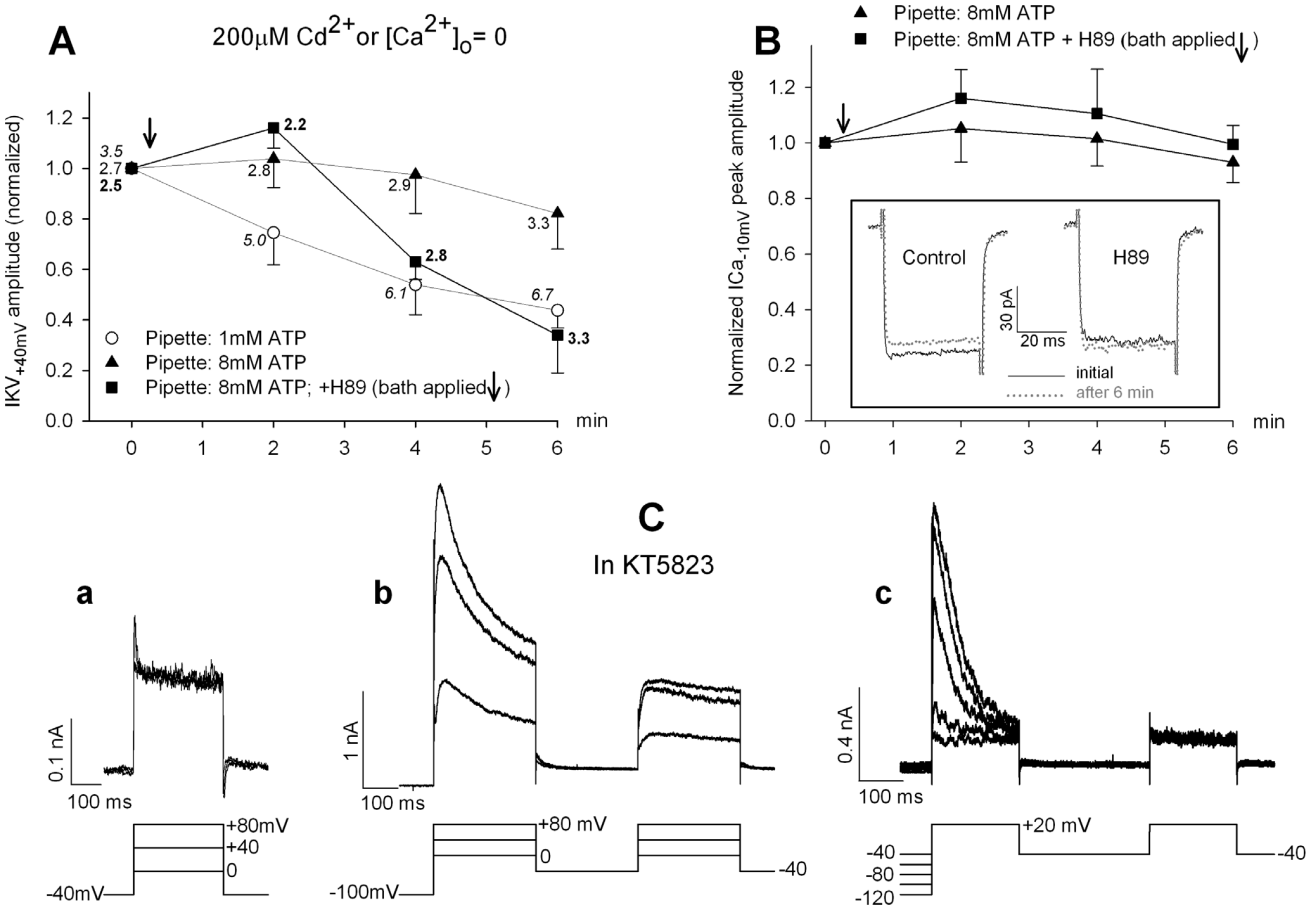
Effect of protein kinase inhibitors on IKV and ICa amplitude. **A.** Isolated IKV are dissected by omitting external calcium or in the presence of 200 µM Cd^2+^ (results were similar and pooled). The mean IKV amplitude (normalized) evoked at +40 mV from a −40 mV holding potential is plotted against time in hair cells recorded with 1 mM ATP pipettes (circles; n = 5), 8 mM ATP pipettes (triangles; n = 7) or with 8 mM ATP pipettes and 10 µM H89 bath-applied at arrow (squares; n = 5). Numbers close to each symbol report the inactivation removal coefficient (*irc*) for IKV following a 1 s conditioning at −100 mV. **B.** Normalized ICa peak amplitude plotted versus time in control cells (triangles; n = 4) or hair cells exposed to 10 µM H89 for 6 min (bath-applied at arrow) (squares; n = 4). Pipette with 8 mM ATP throughout. Representative ICa tracings evoked by −70/−10 mV voltage steps, before and after 6 min in control saline or in the presence of H89, are illustrated in the inset. Tail currents are truncated. **C.** 1 µM KT5823 application for 6 min cancels the voltage dependence of IKD activation when steps start from a holding potential of −40 mV (**a**). Preconditioning the same cell at −100 mV for 1 s (**b**) removes inactivation, and restores activation at the same membrane potentials tested in (**a**). In another cell, preconditioning over the −40/−120 mV range is completely inefficient in removing IKD inactivation (**c**).

The pure IKV component is readily extracted from the IKD complex in [Ca^2+^]_o_ = 0 or by applying external 200 µM Cd^2+^ to block ICa and, consequently, IKCa. Figure 6A illustrates the normalized peak amplitude of IKV over time in the three conditions previously tested for IKD: internal 1 or 8 mM ATP, and internal 8 mM ATP with bath applied 10 µM H89. The corresponding inactivation removal coefficient, *irc*, is reported close to each data point, as evaluated from the absolute current amplitudes. Results mirror the IKD data in indicating that also IKV suffers from a similar rundown (a mean decrease by 57% within 6 min) if not supported by high ATP (low ATP, open circles; n = 5 vs. high ATP, triangles; n = 7), and that it is strongly inhibited by H89 (a mean decrease by 73% after 6 min application, squares; n = 5). Consistent with the observations above for total IKD, the recovery in IKV amplitude due to inactivation removal is impaired by the protein kinase inhibitor, as indicated by constant values of *irc* in the strongly decayed currents. IKV accounts for about 30% of the whole IKD in normal hair cells. Therefore, inactivation of IKV alone does not account for the effects observed for the whole IKD.

The IA dynamics (I–V curve, peak current amplitude, inactivation mechanism) proved remarkably insensitive to the above treatments. Fig. 3C shows that the combined effect of two manipulations, namely 1 mM internal ATP and H89 exposure, which would otherwise strongly depress the IKD complex, does not affect the IA amplitude over time.

The calcium current, ICa, is potentially affected by run-down or (less frequently) by run-up in frog hair cells, unless they are stabilized by high internal ATP [2]. In these conditions, ICa amplitude becomes constant and calcium-dependent inactivation is canceled, even during long lasting current inflow [3]. Figure 6B shows ICa peak amplitude in stabilized hair cells, evoked at −10 mV (where ICa exhibits its maximum) at increasing times in the presence or absence of H89. In the inset of the same figure, representative ICa tracings evoked at −10 mV illustrate that both amplitude and time course of the current are unaffected by time and PK inhibition. ICa appears as the only component of the IKD complex insensitive to protein kinase manipulation by H89.

### Effect of KT5823

The results of H89 application proved highly reproducible in isolated hair cells, in terms of both the time course of the effect and the extent of current decay. KT5823 (1–10 µM, 8 mM ATP in the pipette) gave homogeneous effects on IKD amplitude, similar to those observed with H89. In a group of 5 cells exposed to 1 µM KT5823 in [Ca^2+^]_o_ = 2 mM, the mean amplitude of IKD decreased to 36.8% within 6 min (690.44±95.7 vs. 254.48±120.5 pA; *P*<0.01, paired Student’s *t*-test). In two cells treated with 10 µM KT5823, the decrease was to 44.7%. When external calcium was removed (only IKV surviving), the final mean decrease was to 36.0% of the value before drug application (n = 3). Less predictable were the effects on the inactivation process. In three of these cells (1 µM KT5823) an additional, unexpected impairment of the IKD activation/inactivation mechanism ensued. The IKD complex appeared to lose any voltage dependence in a cell kept at −40 mV holding potential, as if all components with voltage-dependent activation had been canceled by inactivation. A 1-s pre-pulse at −100 mV was sufficient to restore the voltage dependence (Fig. 6Cab). Conversely, in a different cell the inactivation removal was completely inefficient, over the −40/−120 mV range, and the test pulse evoked currents of constant amplitude, independent of previous conditioning (Fig. 6Cc).

### Effect of Inhibitors or Activators

The observations here reported suggest that PK activity is necessary to control the IKD machinery. To test whether the intracellular signaling mechanism(s) might directly involve cAMP or cGMP, the following tools were employed: 300 µM IBMX, a non-specific inhibitor of cAMP and cGMP phosphodiesterases (applied in the bath or via the patch pipette; n = 9); 10 µm Rolipram, a selective cAMP-specific phosphodiesterase (PDE4) inhibitor (n = 3); 500 µM 8-Bromo-cAMP, a cell-permeable cAMP analog, activator of PKA (n = 6); 50–200 µM 8-pCPT-cGMP, a membrane-permeant activator of cGMP-dependent protein kinases and cGMP-gated ion channels (n = 7). Single hair cells were internally perfused via pipettes containing 0–1–8 mM ATP. None of the listed treatments was able to modify the previously described behavior: modifications in IKD current amplitude and inactivation mechanism in low ATP or steady-state conditions in high ATP.

These observations, combined with the data obtained with H89, indicate that adequate availability of ATP and functionality of kinases are both required to maintain the native amplitude of the IKD currents and to remove inactivation of the channels at negative potentials.

### Modulation of Sinusoidal Voltage Commands

**Figure 7:**
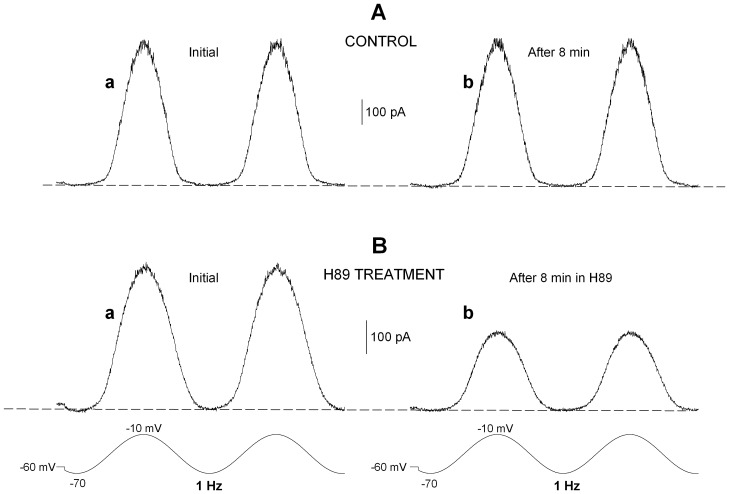
H89 application decreases the total current evoked during sinusoidal hair cell stimulation. Typical tracings of current recorded during −70/−10 mV voltage cycles at 1 Hz in a hair cell at the beginning (**Aa**) and at the end of an 8-min incubation in control conditions (**Ab**) and in a different cell exposed to 10 µM H89 for 8 min (**Bb**) after the initial recording in normal saline (**Ba**). Dashed line indicates zero-current level.

We verified the effects of H89 under conditions which might mimic, more closely than the simple I–V curve, the physiological dynamic behavior of the hair cell during activity. The membrane potential of the hair cell is expected to fluctuate physiologically from a ’resting’ status close to −40 mV (cilia at an intermediate position, a fraction of the receptor transduction channels open), toward −70 mV (all channels closed by the inhibitory deflection of the cupula), or toward a more positive voltage level (activation of additional transduction channels during the excitatory movements of the cupula). The natural membrane potential range of the hair cell might thus span from −10 mV, close to the equilibrium potential of the transduction current, during maximal sensory stimulation, to −70 mV, close to the zero-current voltage, in the absence of any transduction current flow. We thus mimicked the receptor potential time course by driving the hair cell membrane potential with a sinusoidal waveform that fluctuated between −70 and −10 mV, to simulate the whole excitatory/inhibitory cycle of the hair bundle, or between −40 and −10 mV to simulate the excitatory effects only. Figure 7A illustrates the current tracings evoked in the same cell by sinusoidal voltage shifts at 1 Hz in the −70/−10 voltage range in control saline over an 8 min period (8 mM ATP in the pipette). Similarly, Fig. 7B shows the effect of an 8 min exposure to H89 (panel b), compared with the initial tracing in normal saline (panel a). The charge displaced by the voltage shifts was comparable during the two hemicycles of each episode. The total outward charge evoked during a single complete excitatory/inhibitory cycle was used to evaluate the effects. The same experimental conditions as in Table 1 have been tested; the results are partially summarized in Table 2, to show the final result of each treatment.

**Table 2:** Outward charge movement evoked by single depolarizing voltage cycles applied at 1 Hz.

Pipette	In control saline pC	After 8 min pC
	−70/−10 mV cycle −40/−10 mV cycle	−70/−10 mV cycle −40/−10 mV cycle
1 mM ATP	142.9±50.1 199.7±78.4	85.0±42.9¶ 110.1±69.8†
8 mM ATP	111.4±24.6 137.0±30.1	109.5±23.2 129.0±33.3
8 mM ATP+H89*	124.7±29.0 151.1±62.1	50.8±9.4¶ 77.6±30.2†

* 10 µM H89 was added to the bath after the initial measure in control saline. n = 6, in each group. The final vs. initial values in 1 mM and 8 mM ATP+H89 are statistically different (¶ *P*<0.05; † *P*<0.01; paired Student’s *t* test). Mean ± SE.

The same conclusions as in I–V experiments can be readily drawn. Even under these dynamic conditions, the outward potassium current is maintained for several minutes only in the presence of high internal ATP. Low internal ATP or H89 treatment, even in the presence of high ATP, are unable to sustain a stationary potassium outflow, independent of the voltage range explored.

### Current Dissection during Sinusoidal Cycles

**Figure 8:**
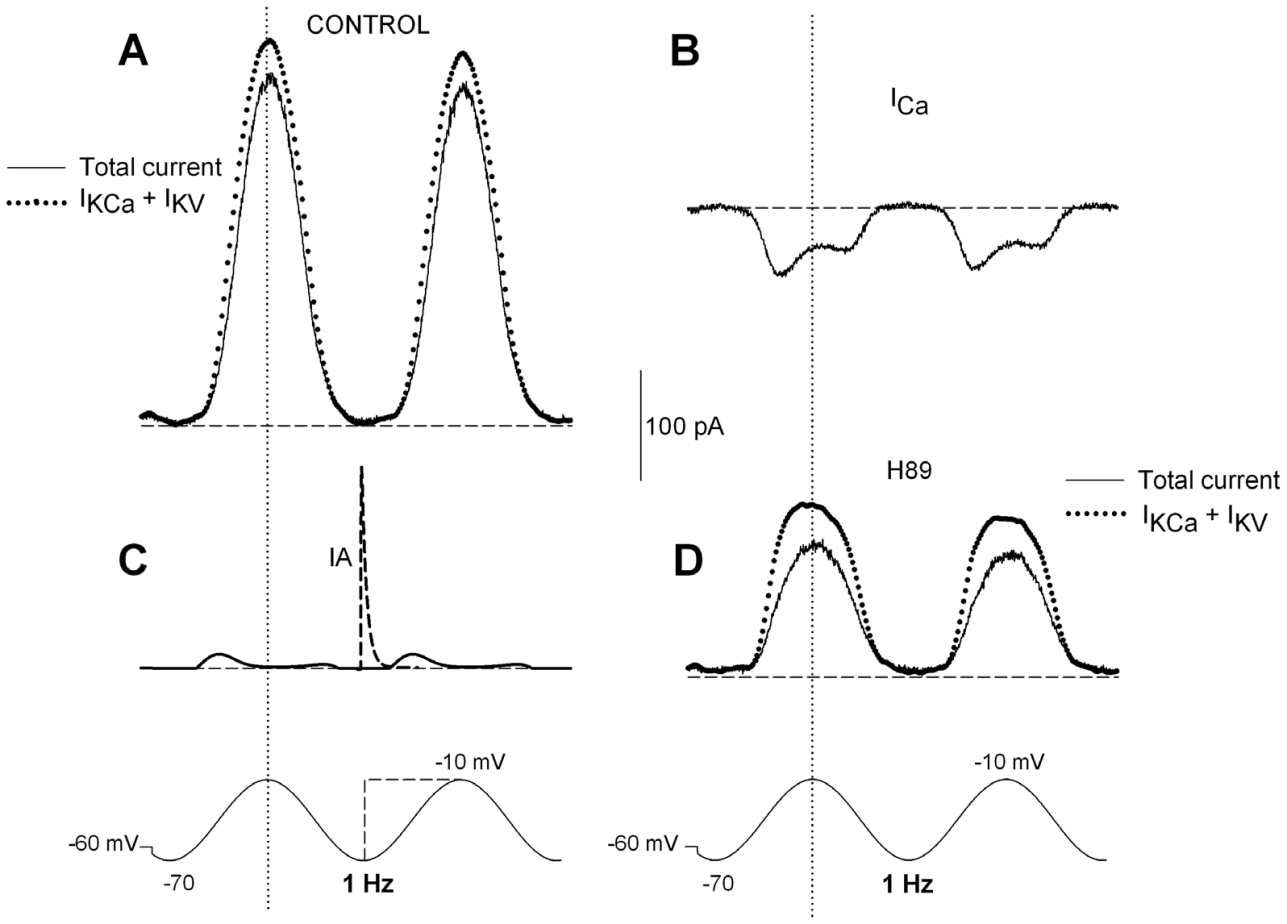
Isolation of the single current components evoked during sinusoidal stimulation in an ideal hair cell, and the effect of H89. **A.** Mean total current from 5 different hair cells recorded during 1-Hz sinusoidal cycles in the −70/−10 mV voltage range, before (continuous line) and after subtraction of the ICa and IA fractions (separately shown in panels **B** and **C**) to dissect the pure IKCa+IKV system (dotted line). **B.** Pure calcium currents recorded from 5 cells during the same stimulation protocol as in **A**. The biphasic behavior of the current during the sinusoidal voltage migration reflects the fact that membrane potential trespasses the peak of the I–V curve for ICa. **C.** IA evoked by the sinusoidal stimulation, as predicted by the mathematical model of the current. During the second cycle, the voltage is instantaneously stepped to the same final level of −10 mV; this fast activation generates a much larger IA (dashed voltage/current tracings). **D.** Mean total current recorded from the same cells as in **A**, after an 8 min treatment with 10 µM H89 (continuous line). Dotted line shows the isolated IKCa+IKV system, after ICa and IA subtraction. Horizontal lines indicate the zero-current level. Vertical lines indicate the symmetry point of each cycle.

As illustrated by Fig. 1, the different components of the potassium current are readily dissected during instantaneous voltage commands at various potentials. The pure calcium current can also be isolated by appropriate ionic manipulations. During slow fluctuations of membrane potential, which are more pertinent to the physiological hair cell behavior, the contributions of the single current components is less directly predictable. Figure 8 illustrates the decomposition of the total outward current in an ideal ‘mean’ hair cell during repeated sinusoidal cycles in the −70/−10 mV voltage range, in control conditions or in the presence of H89. The total current and the pure ICa are derived from direct measurements in patch-clamp (Fig. 8A,B). Since IA cannot be isolated (as was done in Fig. 1) under these conditions, and no selective pharmacological blockers are available, IA contribution was simulated according to the mathematical model described in Methods (Fig. 8C).

The changes in membrane potential here considered are very slow, compared to the time constants of IA activation and inactivation onset/removal, so that the IA conductance does not depart much from its steady-state values, at any potential. Since either the steady-state activation is quite low (below −50 mV) or the steady-state inactivation predominates (above −50 mV), the overall contribution of IA results quite small over the whole cycle (Fig. 8C). Its contribution might obviously be much more relevant for rotations much faster than 1 Hz. This point is illustrated in Fig. 8C where, during the second sinusoidal cycle, voltage is abruptly stepped to the final −10 mV level (dashed current/voltage tracings). The fast current activation now generates a much larger IA component. Although the labyrinthine organs and hair cells are generally looked at as low-frequency mechanical sensors (as compared to their cochlear counterparts), rapid movements of the head might entail components of angular acceleration at quite high frequencies. Under such conditions the contribution of IA might become more relevant in molding the signal that reaches the CNS and the subsequent physiological responses.

Based on the I–V curves reported above, ICa and IA have been considered to be unaffected by H89 during sinusoidal activation as well. The summed IKCa-IKV complex was thus isolated by algebraically subtracting ICa and IA from the total current (Fig. 8D). The decrease of IKCa+IKV amplitude following H89 treatment is evident, as was quantitatively reported in Table 2, and an asymmetry in charge movements between the excitatory and inhibitory hemicycles is revealed, mirroring the two bouts of ICa activation.

## Discussion

The present experiments confirm the presence of a voltage-dependent inactivation mechanism in the delayed potassium current machinery, IKD, in frog hair cells. We show here that the two major potassium currents which build up the IKD complex, IKCa and IKV, exhibit amplitude and steady-state inactivation properties which are drastically influenced by a previously unrecognized phosphorylation-dependent modulation. Such control is independent and specific, for each of the two currents; the transient potassium current, IA (though it exhibits a powerful native inactivation mechanism), and the calcium current, ICa, are completely insensitive to this regulation.

### The KD-conductance Complement of the Hair Cell

The frog hair cell is endowed with a large potassium conductance which could be activated by depolarization, but is made available to the cell in a variable amount, efficiently controlled by the resting membrane potential level. Despite the large variability among cells, in the unstimulated cell maintained at the presumed resting membrane potential around −40 mV, about 4.6 nS of total delayed potassium conductance are available. This figure can be raised by a factor up to 5.5 by progressively removing the fast inactivation, corresponding to the −40 mV status, by a short-lived sojourn (1 s) at more negative potentials; the maximal effect is reached with a conditioning pulse at −120 mV and develops within few milliseconds. Inactivation removal is enhanced by maintaining the cell at hyperpolarized levels for a longer time: the available potassium conductance further increases by an additional factor of 1.6 (+60%), though the process takes several seconds, providing the cell with a total potassium conductance of about 44 nS. The starting conductance level, and the subsequent adjustments due to membrane potential shifts, are all controlled by protein kinase activity. H89 treatment decreases the resting conductance to a mean of 1.4 nS; the available conductance is still sensitive to hyperpolarization, but with a limited ceiling: a 1.7 factor for fast inactivation removal, and an additional 1.16 factor if the slow process of inactivation removal is also allowed to fully develop. The maximal available potassium conductance is now reduced to 2.7 nS (about 6% of the total conductance in control conditions). Normal hair cell activity develops over a voltage range more restricted than that explored by biophysical experiments: it presumably never exceeds −75 mV, the zero-current level below which no cell conductances are active. Still, the reported effects of voltage and protein kinase control are quite relevant even within these functional limits: the KCa-KV conductance available would fluctuate in the 4.6–10.2 nS range under control conditions, and in the 1.4–2.1 nS range following H89 treatment.

For the sake of comparison, the *irc* values (ratio between current amplitude evoked from a conditioning potential that completely removes inactivation and that evoked from the native resting membrane potential), for fast currents that typically exhibit a steady-state inactivation mechanism, are of the same order of magnitude as those here reported for IKD: *irc* = 1.7 for I_Na_ in squid axon, 1.6 for I_Na_ and 4.9 for IA in rat sympathetic neurons [15].

### Protein Kinase Effects

The present experiments aimed at exploring whether kinase activity was implied in the IKD inactivation machinery, but also what kinases and cyclic nucleotides might be involved. We have no definite answer for the latter questions.

As regards the kinase identity, H89 is considered to be specific for PKA, while KT5823 for PKG. The concentrations used here, however, may make the specificity ill-defined. Previous studies have shown that purified PKG can be inhibited by H89 in vitro, although the K_i_ is 10 times higher than that for PKA [16]–[17]. Most data on activation of PKG suggest that its targets are different from those investigated here: nitric oxide inhibited the voltage-dependent ICa in rat vestibular hair cells by the activation of a cGMP-signaling pathway [18], and activation of PKG drastically decreased ICa in hair cells of the frog saccule, via the release of nitric oxide [19].

Few reports in the literature have concerned the mechanism of action of protein kinases on the electrical activity of semicircular canal hair cells. Jagger and Ashmore suggested a regulatory role for protein kinase A in guinea-pig cochlear hair cell [20]–[21]. Their main evidence was based on the fact that bath-applied 8-Bromo-cAMP, an activator of PKA, increased the amplitude of Ikf, a current similar to IKCa.

In the present experiments permeable analogs of cAMP or cGMP, as well as more or less specific phosphodiesterase inhibitors, were apparently unable to influence the final current behavior expected in the presence of different internal ATP levels. This finding could be explained by a significant level of constitutive activation of kinases which maintain the channel protein in a highly phosphorylated state, as occurs in other cell types ([6]; some reviews of the extensive literature: Jonas and Kaczmarek [4]; Schubert and Nelson [22]; Hou et al. [23]).

In the native hair cell the cytosolic millimolar ATP concentration is likely close to that imposed artificially via high-ATP pipettes. Since the basal level of channel phosphorylation would be determined by the equilibrium between phosphorylation and dephosphorylation processes, the use of PK inhibitors would shift such equilibrium toward the dephosphorylated state and account for the decrease in the available potassium conductance and the steady-state inactivation impairment here observed.

### Effect on Current Amplitude

Protein kinase inhibition by H89, and by KT5823, produces two distinct effects: it decreases the amplitude of both IKCa and IKV, and it interferes with their steady-state inactivation mechanism. Both current components are similarly affected, when considered in isolation. This might reflect similar structures of the channel proteins. The IKV fraction contributes to the total IKD by about 30%, so that its depression cannot account for the final cumulative effect on IKD. The involvement of IKCa is demonstrated by considering the difference current before/after Cd^2+^ application: it appears to be intrinsically and genuinely sensitive to kinase inhibition, since no changes are observed in ICa amplitude, which is insensitive to H89 treatment.

Detailed analysis of the effects of H89 on the IKD activation curve (Fig. 5A) is hard, since at least three distinct currents, with different I–V relations, are involved. The steady-state inactivation curve, on the other hand, only reflects the behavior of the two potassium currents (ICa does not exhibit any steady-state inactivation property), which have rather similar molecular structure and display very similar sensitivity to the treatments. Incubation in H89 did not produce any significant shift in the inactivation curve, nor did it have any effect on the slope factor. This would suggest that H89 reduces the number of readily available channels, which maintain their normal responsiveness to depolarization.

### Effect on Steady-state Inactivation Mechanism

A number of voltage-gated channels sustain transient currents. Typically, the sodium and IA currents, once evoked, exhibit a fast decay due to an intrinsic mechanism of fast inactivation (open state inactivation, OSI). The underlying molecular mechanisms are relatively well understood and there is evidence that they are regulated by protein kinase activity: for example, phosphorylation of potassium channels by serine/threonine kinases alters the rate of current decay due to fast inactivation ([4]–[9]; Roeper et al. for Kv1.4 [10]; Jerng et al. for Kv4.2 [8]). For these channel types, when the holding membrane potential is made progressively more negative, the current intensity in response to depolarizing steps to the same voltage levels progressively increases (closed state inactivation, CSI; for a review see Bäring and Covarrubias [24]). The latter principle also applies to IKCa and IKV in frog hair cells; it is indicated here with the more traditional note ‘steady-state inactivation’. Contrary to OSI, however, the molecular basis of CSI is not fully understood. A conceptual model, originally proposed for spHCN channels, assumes that CSI may result from a temporary separation of the voltage sensor and the activation gate [25]. Analysis of these structural channel modifications are beyond the purpose of this paper, which remains mainly phenomenological. The novel observation, however, is that phosphorylation-mediated effects are able not only to influence the open state or the number of responsive potassium channels, but also to control, in a voltage-dependent manner, the availability of the closed channels to subsequent depolarizations. This is particularly evident in the case of the KT5823 treatment, which in some cases has canceled any voltage-dependent inactivation removal, resulting in an irreversible channel blockade, both at membrane potential values at which they are physiologically closed and in response to depolarization, which should move them to the open state. An important point that emerges from these experiments is that the mechanisms that control the amplitude of the evoked currents or the removal of inactivation are independent processes, differently affected by protein kinase manipulation. Were this not the case, the *irc* values would always mirror the maximal amplitude of the evoked responses.

### A Physiological Role for the IKD Inactivation Mechanism?

The kinetics of onset and removal of IKD inactivation are in the order of milliseconds, so that any shift of the hair cell momentary potential (action of the efferent systems, modifications of the tonic transduction current, fluctuations of the ionic gradients…) that occurred during the ongoing cell activity would produce a corresponding adjustment of the largely variable KD conductance.

Voltage steps and sinusoidal voltage shifts simulating the ciliary excitatory/inhibitory deflections of the hair cell clearly illustrate the role of the single current components evoked by the transduction current. The transient role of IA is expected to contribute only in the presence of strong, rapid potential fluctuations, while the ongoing hair cell activity is presumably controlled by the IKCa and IKV components, which counteract the sensory transduction current in setting the momentary membrane potential of the cell. The calcium current does not exhibit any intrinsic regulatory mechanism, so that its amplitude, and the related cytoneural synaptic activity, is exclusively governed via its voltage dependence properties. Conversely, IKCa and IKV channels are controlled in a more complex way in frog hair cells. Their variable phosphorylation level, inhibited by H89 or by KT5823, might thus operate to continuously adjust the balance between the depolarizing transducer current and the repolarizing potassium current, thus setting the calcium inflow, and indirectly the transmitter release rate at the cytoneural junction, which ultimately generates the sensory information to be conducted to the central nervous system.
